# Supplementary material for: Optimizing Messenger RNA Analysis Using Ultra-Wide Pore Size Exclusion Chromatography Columns
Source: Int J Mol Sci. 2024 Jun 6;25(11):6254. doi: 10.3390/ijms25116254 (PMC11172508; doi:10.3390/ijms25116254)
Supplement: Supplementary file 1 [file ijms-25-06254-s001.zip › ijms-3018918-supplementary.pptx]

## Slide 1
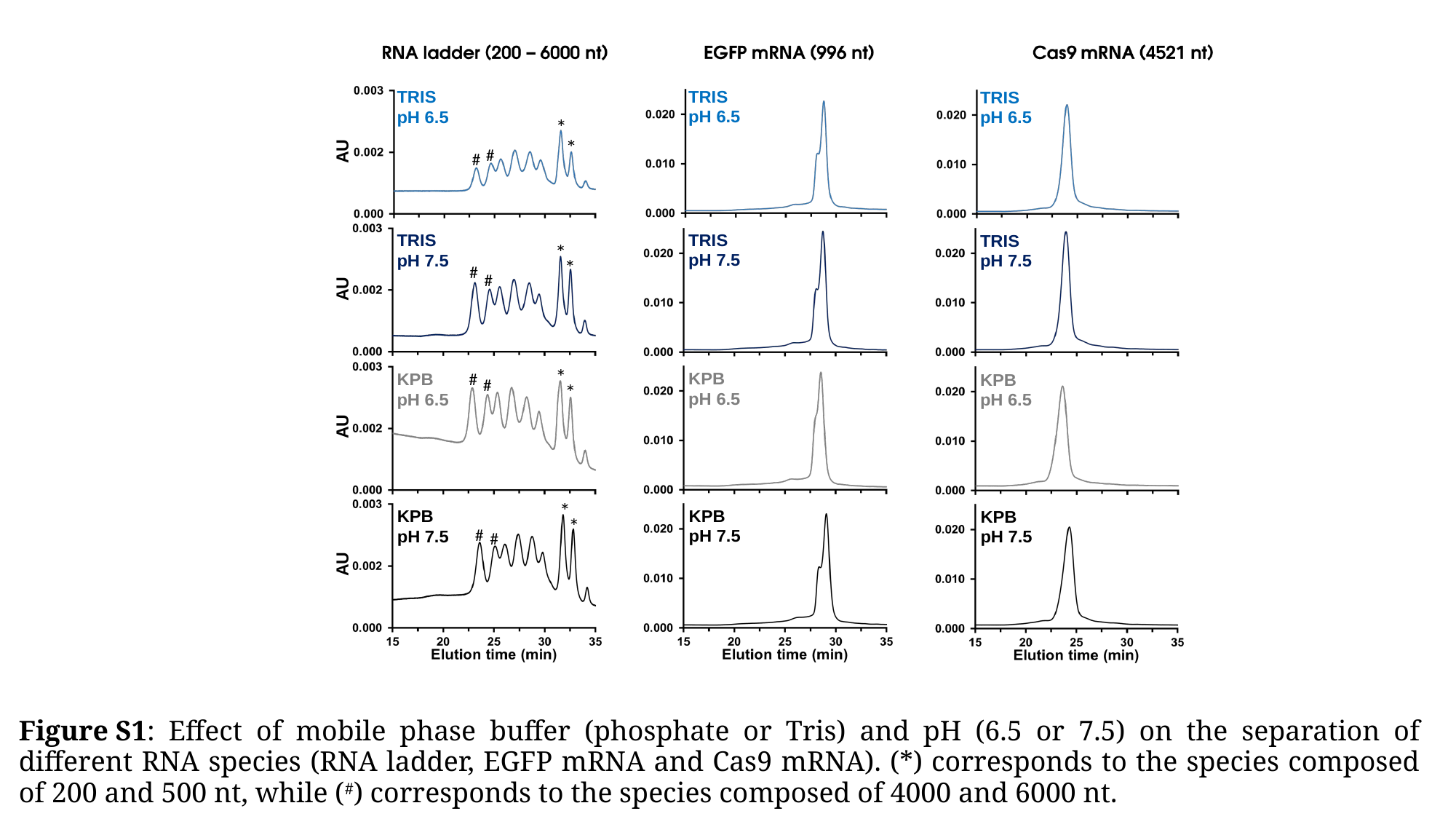

Figure S1: Effect of mobile phase buffer (phosphate or Tris) and pH (6.5 or 7.5) on the separation of different RNA species (RNA ladder, EGFP mRNA and Cas9 mRNA). (*) corresponds to the species composed of 200 and 500 nt, while (#) corresponds to the species composed of 4000 and 6000 nt.

## Slide 2
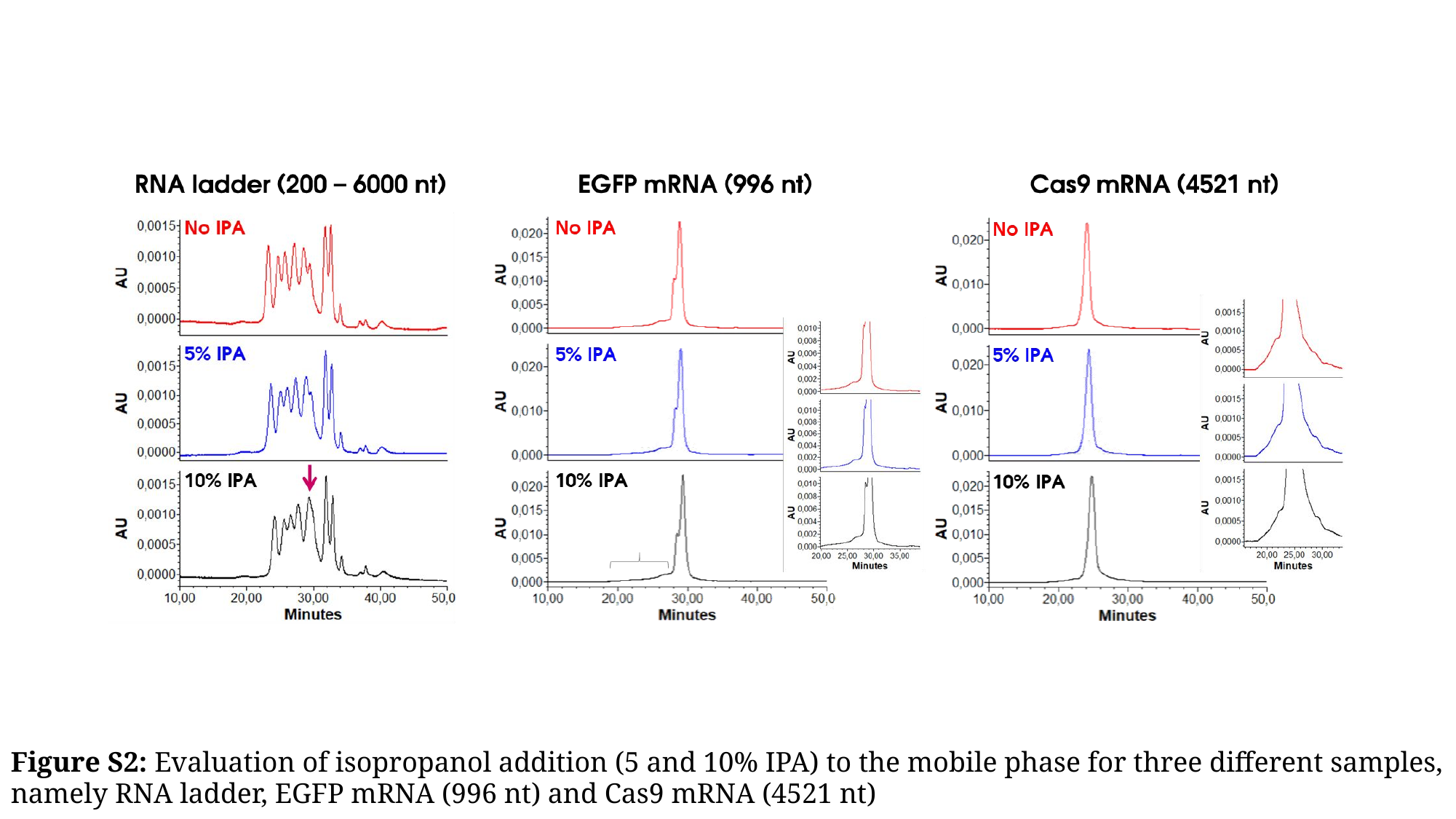

Figure S2: Evaluation of isopropanol addition (5 and 10% IPA) to the mobile phase for three different samples, namely RNA ladder, EGFP mRNA (996 nt) and Cas9 mRNA (4521 nt)

## Slide 3
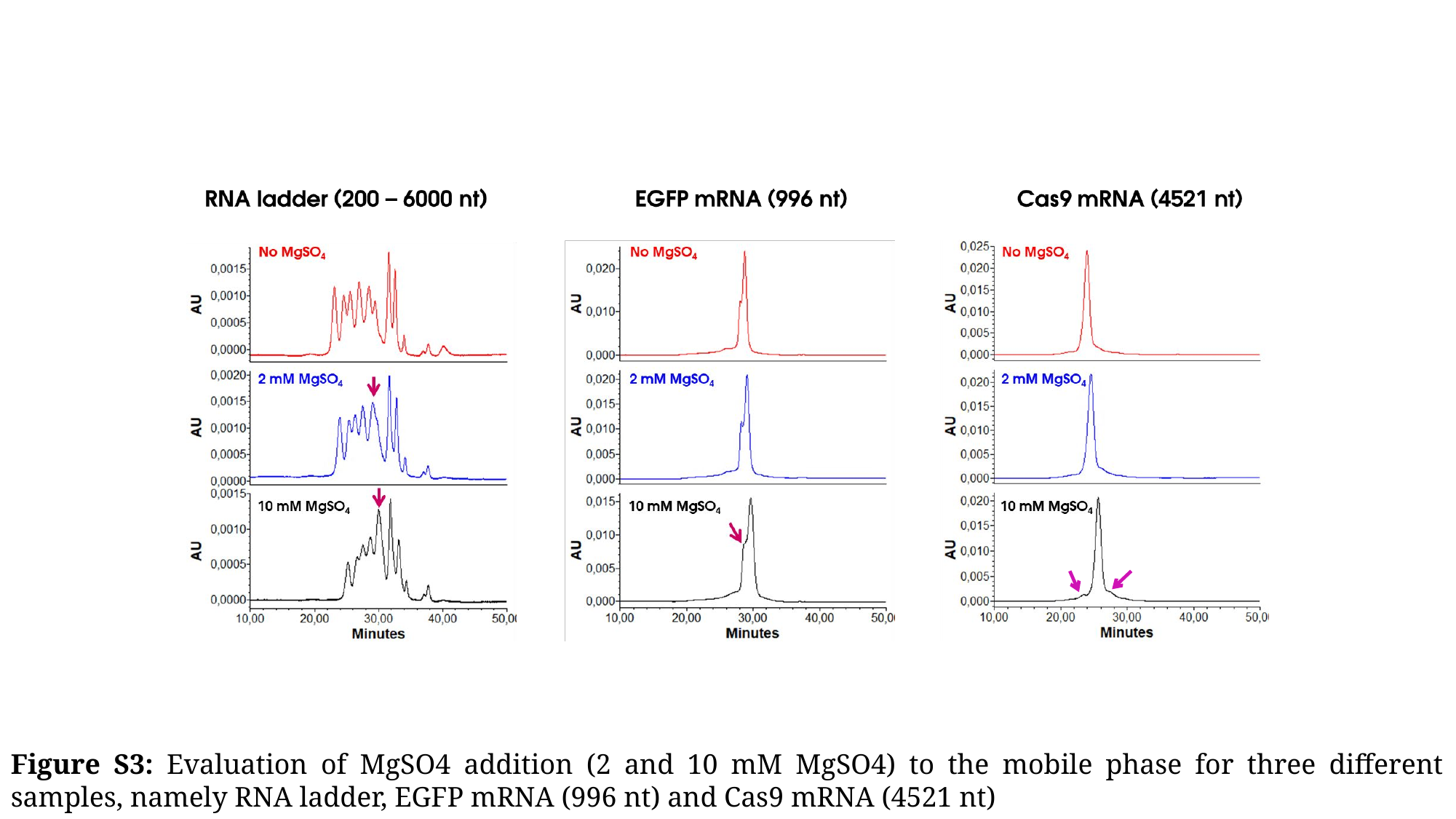

Figure S3: Evaluation of MgSO4 addition (2 and 10 mM MgSO4) to the mobile phase for three different samples, namely RNA ladder, EGFP mRNA (996 nt) and Cas9 mRNA (4521 nt)

## Slide 4
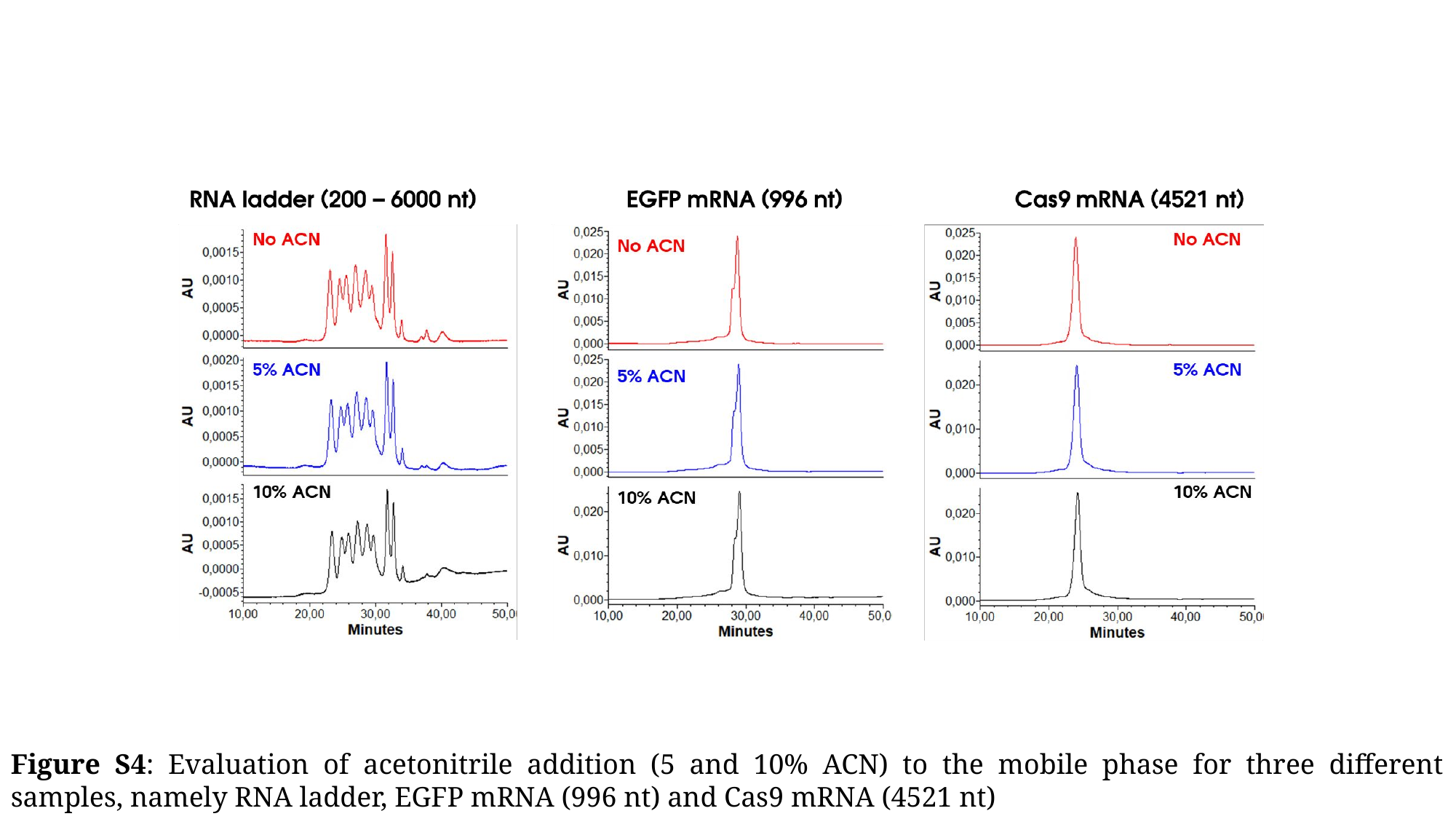

Figure S4: Evaluation of acetonitrile addition (5 and 10% ACN) to the mobile phase for three different samples, namely RNA ladder, EGFP mRNA (996 nt) and Cas9 mRNA (4521 nt)

## Slide 5
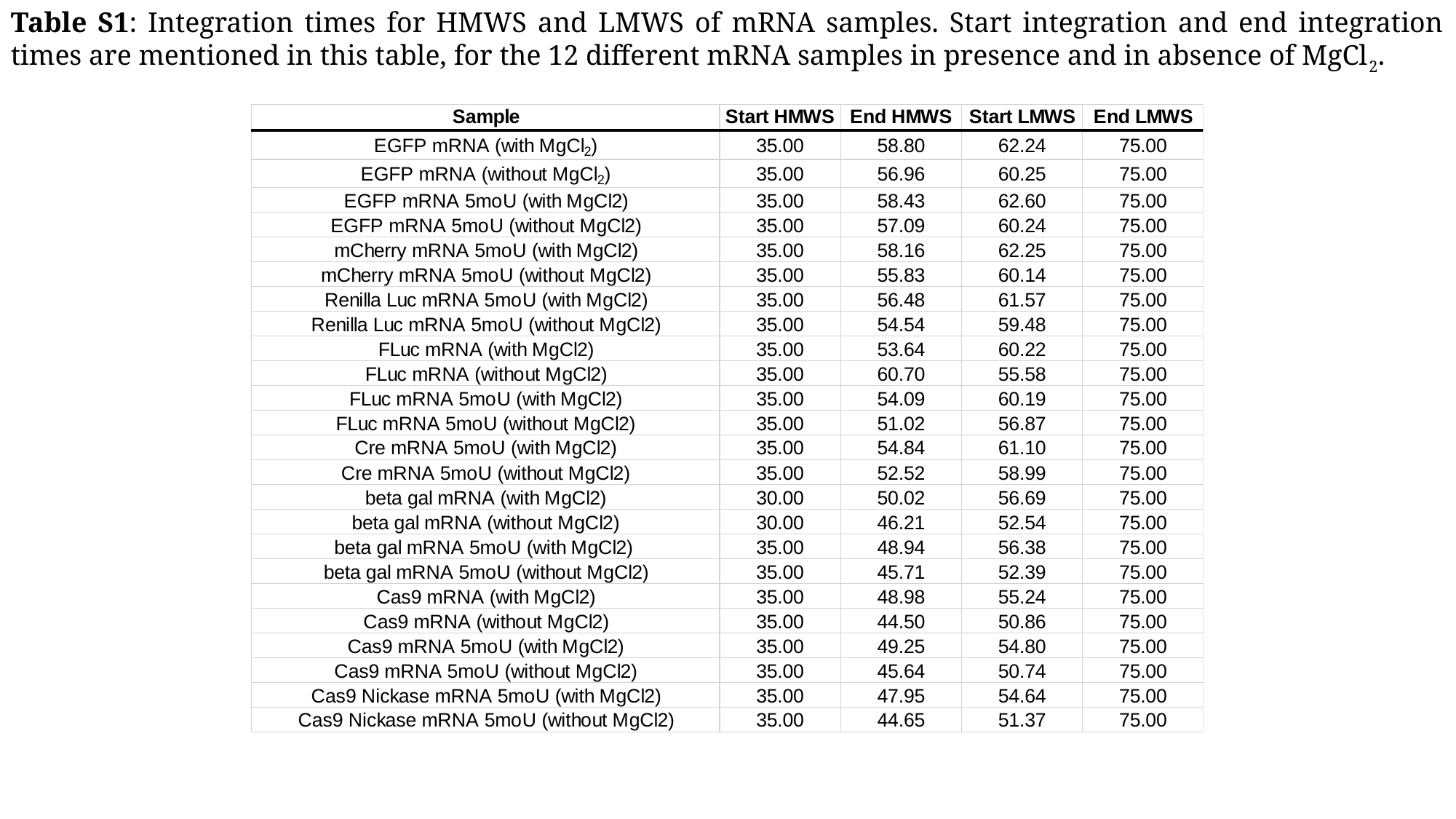

Table S1: Integration times for HMWS and LMWS of mRNA samples. Start integration and end integration times are mentioned in this table, for the 12 different mRNA samples in presence and in absence of MgCl2.
